# The Role of Antioxidant Transcription Factor Nrf2 and Its Activating Compounds in Systemic Lupus Erythematosus

**DOI:** 10.3390/antiox13101224

**Published:** 2024-10-11

**Authors:** Lu Liu, Karina de Leeuw, Harry van Goor, Johanna Westra

**Affiliations:** 1 Department of Rheumatology and Clinical Immunology, University Medical Centre Groningen, University of Groningen, 9713 GZ Groningen, The Netherlands; 2 Department of Pathology and Medical Biology, University Medical Centre Groningen, University of Groningen, 9713 GZ Groningen, The Netherlands

**Keywords:** systemic lupus erythematosus, lupus nephritis, Nrf2 pathway, oxidative stress, treatment

## Abstract

Systemic lupus erythematosus (SLE) is a complex autoimmune disease in which kidney involvement, so-called lupus nephritis (LN), is common and one of the most severe manifestations. Oxidative stress (OS) may play a role in the pathogenesis of LN through the exacerbation of inflammation and immune cell dysfunction/dysregulation. Nuclear factor erythroid 2-related factor 2 (Nrf2), also known as nuclear factor erythroid-derived 2-like 2, is a transcription factor that in humans is encoded by the NFE2L2 gene and is regarded as a central regulator of the antioxidative response. Nrf2-activating compounds have been shown to alleviate oxidative stress in cells and tissues of lupus-prone mice. Although the precise mechanisms of Nrf2 activation on the immune system in SLE remain to be elucidated, Nrf2-activating compounds are considered novel therapeutical options to suppress OS and thereby might alleviate disease activity in SLE, especially in LN. This review therefore summarizes the role of the Nrf2 signaling pathway in the pathogenesis of SLE with LN and describes compounds modulating this pathway as potential additional clinical interventions.

## 1. Introduction

Systemic lupus erythematosus (SLE), a complex chronic autoimmune disease involving innate and adaptive immune dysregulation, is accompanied by a wide range of clinical–pathological manifestations such as fever, rash, arthritis and internal organ involvement [1]. SLE is characterized by increased production of autoantibodies against nuclear components, defective clearance of immune complexes and loss of immune tolerance [2]. Lupus nephritis (LN) is one of the most severe organ manifestations of SLE, affecting approximately 20–40% of patients [3]. Patients with LN 10–30% will progress to end-stage renal disease (ESRD) within 10 years, resulting in increased morbidity and mortality [4,5]. While better detection and advanced treatment of SLE can help to diagnose the disease in an early stage and ameliorate the 10-year survival rate, there is still room for improvement of end-organ involvement and mortality [6]. Until recently, treatment for SLE was based on conventional treatments such as hydroxychloroquine, corticosteroids and/or classical immunosuppressants [7]. However, today, biologicals such as belimumab and anifrolumab can be prescribed [7]. Also, the long-term use of immunosuppressive agents can cause a variety of harmful adverse effects [8]. Therefore, nonpharmacological interventions such as vitamin D supplementation, dietary modifications and avoiding ultraviolet exposure are important as well [9].

The pathogenesis of SLE is complex, but one of the main features is the formation of autoantibody-mediated immune complexes (IC). In the case of LN, this IC deposit in glomeruli, resulting in a local cascade of inflammatory reactions, including complement activation. Activation of neutrophils and neutrophil extracellular trap (NETs) formation occur as well [10]. Recently, many studies demonstrated that oxidative stress (OS) is also an important player in the pathogenesis of SLE and might therefore be a potential therapeutic target [11]. OS involves moderate-to-high levels of oxygen/nitrogen radicals and non-radical reactive substances (collectively referred to as reactive oxygen species, reactive nitrogen species and reactive sulfur species (ROS/RNS/RSS)). This has been described within the framework known as the ‘Reactive Species Interactome (RSI)’, a multilevel redox regulatory system that efficiently senses and adapts to environmental changes and stressors [12,13]. RSI in regulating physiological processes is termed “eustress”, which is characterized by oxidants involved in the modulation of various biochemical transformations, including processes related to nuclear factor erythroid 2-related factor 2 (Nrf2) [14]. Conversely, the excessive production of oxidants and reduced clearance of oxidants leads to harmful conditions, known as “oxidative stress” [14]. Under the equilibrium between the production and clearance of ROS, ROS may act as a regulatory medium in various processes, for instance, in blood pressure regulation, immune responses, metabolism and mitochondrial physiology [15]. ROS-mediated reactions probably establish “redox homeostasis” without causing OS-induced cell damage. However, under exposure to endogenous and exogenous toxins, continuous accumulation of ROS leads to cascading reactions and, consequently, “distress” (an excessive and toxic oxidative burden) [16]. The bidirectional relationship between OS and an activated immune response has been widely studied as an emerging paradigm in the pathogenesis of SLE [17]. In addition, ROS/RNS/RSS and ROS/RNS/RSS-derived products are detected in kidney biopsies from patients with LN. Also, markers of OS are more present in the serum and urine of SLE patients [18,19].

Transcription factor Nrf2 is considered to play a central role in cellular metabolic events involved in antioxidant and anti-inflammatory defenses. Several studies have suggested that Nrf2 activation has the potential to alleviate OS damage in kidneys and to improve renal function [20]. Also, Nrf2 knockout mice are more prone to exhibiting lupus-like autoimmune nephritis than wild-type mice [21]. New options for SLE therapy are being developed, and regulating Nrf2 and its downstream effects appears to be a possible approach to improve renal function, and as such may have an additional role in the treatment of SLE and especially LN. 

This review describes the role of Nrf2 in SLE, especially in LN, considering pathogenic mechanisms in mice models as well as clinical studies. Moreover, we provide an overview of the current and upcoming compounds that potentially are an effective therapy for activating Nrf2 in patients and mice models. Finally, the current nutritional interventions targeting this pathway will be discussed.

## 2. Nrf2/Keap1 Signaling Pathway

**Figure 1:**
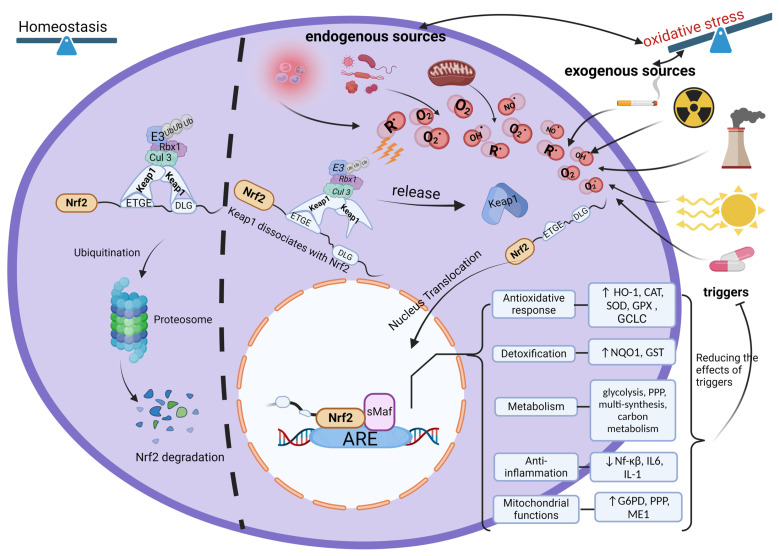
The biological functions of the Nrf2 pathway and interaction with oxidative stress.

In 1994, Nrf2 was first identified as the Cap’n’Collar (CNC) subfamily of basic leucine zipper (bZIP) transcription factors that bind to a β-globin locus control region and has been shown to regulate anti-oxidative responses, the activation of detoxification enzymes, and reactive protein oxygen-scavenging enzymes [22]. Beyond these, Nrf2 is involved in numerous other cellular processes, including the regulation of inflammation, metabolism and cancer prevention [23]. It is known that Nrf2 contains seven highly conserved Nrf2–ECH homology (Neh) domains, Neh1 through 7, with different transcriptional functions [24]. From these, Neh1 and Neh2 are most important: Neh1 is a DNA-bending domain containing the CNC-bZIP region, in which the amino acid sequence is highly conserved throughout multiple species, playing a crucial role in mediating small musculoaponeurotic fibrosarcoma (sMaf), binding to antioxidant responsive element (ARE) and regulating transcription factor activity [24,25]. Neh2 domain is a redox-sensitive degron, containing two conserved amino acids, DLG and ETGE, mediating the negative interaction of Kelch-like ECH-associated protein 1(Keap1), associated with Nrf2, inducing ubiquitination of Nrf2 and subsequent proteasomal degradation [24,25]. Under cellular homeostasis conditions, the abundance of Nrf2 is low as a result of its binding with Keap1 (an adapter protein that assembles with Cullin 3-based E3 ubiquitin ligase (Cul-3)), which mediates Nrf2 degradation via the ubiquitin–proteasome system, thereby preventing Nrf2 translocation into the nucleus [26]. However, under OS conditions, the degradation of Nrf2 is hindered since cysteine modifications alter the conformation of Keap1, leading to weakened binding affinity between Keap1 and DLG on Nrf2 motif, preventing Nrf2 ubiquitination. Surviving Nrf2 translocates into the nucleus where it binds to ARE sequences in the DNA assisted by sMaf. Subsequently, Nrf2 triggers the transcription of detoxification enzymes, antioxidants and metabolic and anti-inflammatory response proteins, increasing expression levels amongst others of NAD(P)H quinone oxidoreductase (NQO1), superoxide dismutase (SOD), catalase (CAT), glutathione peroxidase, thioredoxin and heme oxygenase-1 (HO-1) and enhancing antioxidant enzyme activities (see Figure 1) [25,26,27].

Endogenous (such as inflammation, pathogens, viruses, mitochondrial respiration chain, etc.) and exogenous (pollution, UV light, radiation, drugs, toxins, smoking, etc.) factors can contribute to the generation of free radicals, and excessive free radicals lead to oxidative stress. In homeostasis conditions, there is Nrf2 degradation when the inhibitor Keap1 binds to Nrf2 while under oxidative stress conditions, Nrf2 is activated through disassociating with Keap1 and is translocated to the nucleus; after that, Nrf2 binds to ARE combined with the sMAF protein, promoting the activation of a variety of target genes. Oxidative stress is inhibited as a response; ↑, upregulation; ↓, downregulation.

Abbreviations: ARE, antioxidant responsive element; Keap1, Kelch-like ECH-associated protein 1; Cul3, Cullin 3; Nrf2, nuclear factor-erythroid 2-related factor; sMAF, small musculoaponeurotic fibrosarcoma proteins; Eb, ubiquitin moiety; GCLC, glutamate–cysteine ligase catalytic; NQO1, NADPH quinone oxidoreductase enzyme; SOD, superoxide dismutase; HO-1, heme oxygenase-1; CAT, catalase; GPX, Glutathione peroxidase; GST, glutathione S-transferase; G6PD, glucose-6-phosphate dehydrogenase; PPP, pentose phosphate pathway; ME1, malic enzyme 1, NF-κB, nuclear factor-kappa-light-chain-enhancer of activated B cells; IL-6, interleukin 6; IL-1, interleukin 1

### 2.1. Interaction between Nrf2 and NF-κB Pathways

In the past decades, many studies have shown that Nrf2 plays an important role in diseases that are affected by OS and inflammation, including autoimmune diseases, inflammatory bowel disease, hypertension and neurological diseases [28,29,30]. OS has been reported to play a role in many other kidney diseases as well [31]. The kidney is a highly metabolic organ, which makes its DNA, proteins and lipids of tissue cells vulnerable to damage by ROS, which accelerates damage progression in the glomeruli, renal tubules and renal interstitium, as well as results in the disappearance of foot processes on podocytes and apoptosis of podocytes [32,33,34]. Additionally, OS aggravates the release of biomarkers and inflammatory mediators from vascular endothelial cells. Eventually, this leads to glomerular damage, tubular atrophy and interstitial fibrosis, as well as to the progressive decline in renal function and renal failure [19]. For example, one study has identified 41 OS-related differentially expressed genes associated with LN pathogenesis, including STAT1, SETX, PRODH and TXN2. Additionally, B cells, T cells, dendritic cells and activated natural killer cells were involved, revealing the intricate interplay between OS and immune activation in LN [35]. Furthermore, lupus animal experiments have demonstrated that inducing HO-1 or inhibiting the phosphatidylinositol 3-kinase (PI3K)/mammalian target of the rapamycin (mTOR)-mediated pathway may potentially reduce RSI expression, leading to the amelioration of LN [36,37].

**Figure 2:**
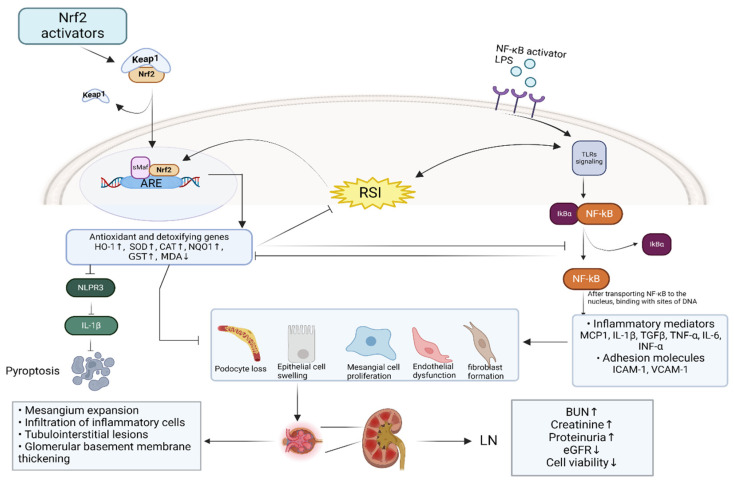
The depiction of the crosstalk of the Nrf2 and NF-κΒ in LN. ↑, upregulation; ↓, downregulation.

OS-induced inflammation is mainly caused by nuclear factor-kappa B (NF-κB) activation. In addition, the interplay between the Nrf2 and NF-κB pathways is sophisticated, and they affect each other through different mechanisms [38]. NF-κB is a key transcription factor that rapidly regulates cellular responses and processes such as inflammation, immune response, apoptosis, cell growth and acute phase responses [38]. In a quiescent state, NF-κB dimers bind with IκB, the negative regulator of NF-κB, to prevent the activation of NF-κB. The most common member of IκB in mediating NF-κB pathway activation is IκBα. In response to an array of stimuli that activate Toll-like receptors (TLRs, belonging to the pattern recognition receptor family), B cell receptors and T cell receptors, the IκBα protein is degraded and undergoes specific ubiquitination, phosphorylation and proteasomal degradation, leading to the release and translocation of NF-κB dimers to the nucleus, where they bind to specific DNA sequences, which initiates the transcription of inflammatory genes [38,39,40]. In LN, NF-κB activation increases the expression of pro-inflammatory cytokines and chemokines such as tumor necrosis factor-alpha (TNF-α), transforming growth factor-beta (TGF-β), interleukin-1beta (IL-1β), interleukin-6 (IL-6), monocyte chemoattractant Protein-1 (MCP-1) and interferon-alpha (IFN- α), and induces an expression of adhesion molecules in epithelial cells such as intercellular adhesion molecule 1 (ICAM-1) and vascular cell adhesion molecule 1 (VCAM-1) [41,42]. Notably, NLRP3 inflammasome is another pathway by which Toll-like receptor 4 (TLR4) and NF-κB activation induce inflammation and apoptosis [43]. The anti-inflammatory effects of Nrf2 may be also related to its inhibitory role in NLRP3 inflammasome activation by inhibiting NF-κB [44]. Many studies have supported the fact that OS and inflammation are mutually dependent and interconnected processes. Inflammatory cells produce large amounts of ROS at the inflammatory site, causing increased oxidative damage, while OS products enhance pro-inflammatory responses. Overall, Nrf2 plays a key role in cellular detoxification, antioxidation and anti-inflammatory effects in kidney diseases [45]. In Figure 2, a schematic representation is shown of the crosstalk between Nrf2 and NF-κB in LN.

Nrf2 is pivotal in the regulation of redox homeostasis, as it has an indirect interaction with NF-kB activity via various signaling pathways. Lipopolysaccharide (LPS) simultaneously activates a fast, proinflammatory NF-κB response and a slow Nrf2 response. The NF-κB response is subsequently inhibited when Nrf2 is active. Moreover, both NF-κB and Nrf2 transcription factors require the coactivator CBP/p300, which is a histone acetyltransferase that acetylates and increases DNA-binding capacity. Therefore, NF-κB overproduction impedes the availability of CBP/p300 for Nrf2, hence reducing its transcriptional capacity, whereas NF-κB knockdown shows the opposite effect [46]. 

Abbreviations: LN, lupus nephritis; LPS, lipopolysaccharide; RSI, Reactive Species Interactome; ARE, antioxidant responsive element; Keap1, Kelch-like ECH-associated protein 1; Nrf2, nuclear factor-erythroid 2-related factor; sMAF, small musculoaponeurotic fibrosarcoma proteins; SOD, superoxide dismutase; HO-1, heme oxygenase-1; CAT, catalase; NQO1, NADPH quinone oxidoreductase enzyme; GST, glutathione S-transferase; MDA, malondialdehyde; NLPR3, NLR family pyrin domain containing 3; NF-κB, nuclear factor-kappa-light-chain-enhancer of activated B cells; IkBα, nuclear factor of kappa light polypeptide gene enhancer in Bcell inhibitor alpha; IL-1β, onterleukin 1 beta; TLRs, Toll-like receptors; MCP-1, monocyte chemoattractant protein 1; TGFβ, Transforming growth factor beta; TNF-α, Tumor necrosis factor-alpha; IL-6, interleukin 6; INF-α, Interferon alpha; ICAM-1, Intercellular Adhesion Molecule 1; VCAM-1, vascular cell adhesion molecule 1; BUN, Blood urea nitrogen; eGFR, estimated Glomerular filtration rate; BUN, Blood urea nitrogen

### 2.2. Nrf2 Expression and Activation in Animal Models of LN

The first evidence that showed the potential role of Nrf2 deficiency in susceptibility for SLE in MRL/Mp-lpr/lpr (MRL/lpr) lupus mice was published in 2001 by Yoh et al. [21]. A shortened lifespan (all mice died within 25 months with severe glomerular lesions), increased the influx of lymphocytes and macrophages in the renal interstitium, and slight splenomegaly were detected in female Nrf2−/−mice. In addition to this, lipid peroxidation levels were increased in Nrf2−/−female mice, indicating increased OS. In a follow-up study, unexpectedly, they showed that fifty percent of Nrf2−/−lpr/lpr female mice had longer survival times than Nrf2+/+lpr/lpr female mice [47]. Decreased immune complex deposits, a reduction of infiltrated cells and fewer renal injuries were observed in Nrf2-deficient lpr female mice. Moreover, lymphadenopathy was suppressed, and immunologic abnormality was improved via increasing TNF-α-mediated apoptosis through glutathione (GSH) depletion, speculating that Nrf2 deficiency enhanced TNF-α-mediated apoptosis, which coincidentally alleviated the accelerating effect of autoimmune disease in these mice. However, in general, Nrf2 activation may prevent the progression of LN through the inhibition of oxidative injury and by negatively regulating the NF-κB pathway via signaling through TLR4 and the expression of high-mobility group box 1 (HMGB1) [48,49]. Moreover, the activation of Nrf2 resulted in shifting M1-like macrophages towards M2-like macrophages and lowered the interferon signature in pristane-treated mice [50]. In another experimental model with Nrf2−/−B6/lpr LN-prone mice, it was shown that Nrf2 deficiency changed the proportion of Th17 cells and expression of Th17-related genes, thereby altering relevant cytokines [51]. Furthermore, Nrf2 deficiency in experimental animal models displayed reduced antioxidant capacity, increased oxidative tissue lesions [51,52] and increased lupus-like autoimmune nephritis [21].

Recently, a wide range of Nrf2 activators have been used to explore the therapeutic potential of Nrf2 in experimental LN models. Nrf2 activators can act through different therapeutic targets via mechanisms generally categorized as follows: (i) small molecule compounds targeting Nrf2-associated protein–protein interactions; (ii) compounds that individually or in combination modify different cysteine residues on Keap1, inducing conformational changes that prevent Nrf2 ubiquitination; (iii) activators that enhance the stability of Nrf2 by regulating its phosphorylation, indirectly activating Nrf2 [51,53,54]. Many studies that investigated Nrf2-activating compounds in LN mice models are summarized in (Table 1). In general, these studies demonstrated that Nrf2 activation led to decreased OS, reduced inflammation and improved renal function in mice models. Taken together, all these findings demonstrated that Nrf2 may have a nephroprotective role in SLE, suggesting that activating Nrf2 could be a promising additional therapeutic target for the treatment of LN [51,52,55].

### 2.3. Nrf2 Expression and Activation in SLE with LN

As shown from the mice model studies and discussed by Nezu et al., alleviating OS by Nrf2 activation may be a feasible strategy for treating autoimmune nephritis [70]. However, there are not many clinical studies on the involvement of Nrf2 in SLE. An analysis of the human Nrf2 gene structure in a small number of patients indicated that polymorphisms in the promoter and regulatory regions of the Nrf2 gene were not significantly associated with the risk of developing SLE [71]; however, the Nrf2-653 G/A polymorphism has been associated with the development of nephritis in childhood-onset female SLE patients [72].

Some studies investigated the expression of Nrf2 in different cells or structures. For example, it has been shown that Nrf2 and its downstream target genes NQO1 and 8-oxo-7,8-dihydro-2′-deoxyguanosine were activated in glomeruli in LN patients, and an increased expression of Nrf2 has also been observed in glomerulonephritis and in acute kidney injury [48,73]. Gautam et al. demonstrated that impaired Nrf2 expression resulted in increased ROS in dendritic cells in patients with high SLEDAI [74]. Another study analyzing T and NK cell subsets in SLE patients showed that OS was increased along with variations in the intensity of Nrf2, Keap1 and HO-1 expression across different cell subsets, and that Keap1 expression was positively correlated with SLEDAI in most T and NK cell subsets [75]. In SLE peripheral blood, the expression of Nrf2 was low in leukocytes compared to the healthy control (HC) [50]. Another study in LN patients revealed a positive correlation between the estimated glomerular filtration rate (eGFR) and SLEDAI with Nrf2 serum protein levels. Furthermore, increased Nrf2 protein was seen in glomeruli and tubular mesangium [76]. An in vitro study showed that in the peripheral blood mononuclear cells (PBMCs) of SLE patients stimulated with a Nrf2 agonist, cellular lipid peroxidation was decreased, while mRNA expression of HO-1, NQO1 and GCLM was enhanced, showing an induced anti-oxidative response [77]. Nrf2 may also play a role in inhibiting neutrophil activation and NETosis formation. Rysenga et al. found that Nrf2 activation inhibited stimulus-induced NETosis and ROS formation in neutrophils while upregulating the expression of Nrf2 [78].

In summary, with the scarce current knowledge on Nrf2 in SLE patients, it seems that moderately up-regulating Nrf2 can activate anti-inflammatory and antioxidant systems, which might exert a protective effect on LN [29]; therefore, it could provide a new additional therapeutic strategy for SLE.

## 3. Therapeutic Approaches in SLE with LN via Modulation of Oxidative Stress

**Figure 3:**
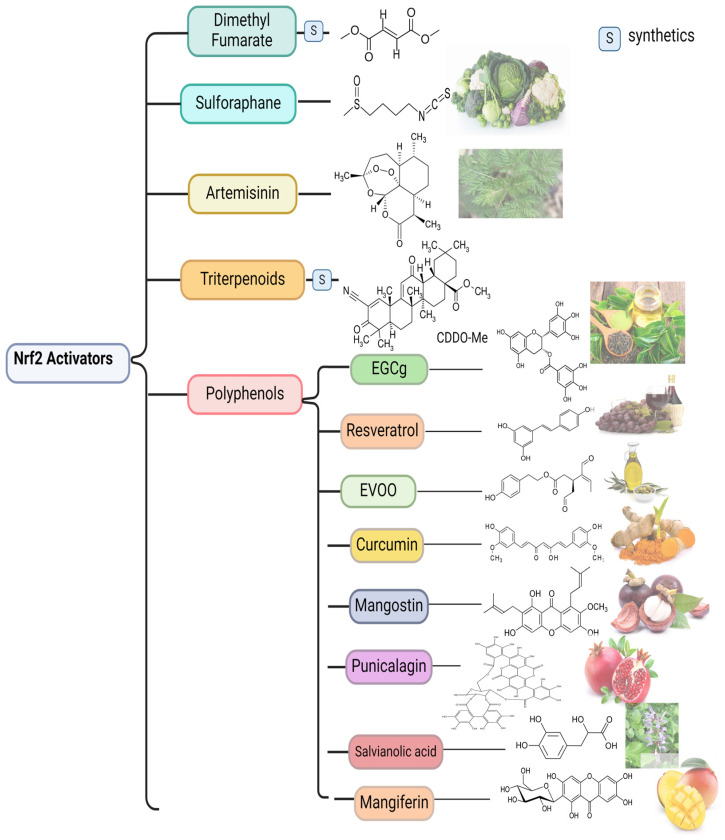
Current Nrf2 activators described in SLE.

In recent years, some studies have revealed that natural products derived from animals or plants might have beneficial effects on disease activity in SLE by influencing OS. In the next part of this review, different compounds that activate Nrf2 and thereby reduce OS are discussed (Figure 3).

Of all the activators, the chemical formulas and sources are shown. Those with an “S” in the formula are synthetic compounds. Without showing sources, the images at the end are synthetic compounds.

Abbreviations: EGCG, Epigallocatechin-3-gallate; EVOO, extra virgin olive oil.

### 3.1. Dimethyl Fumarate

Dimethyl fumarate (DMF) is a methyl ester of fumaric acid esters (FAEs) that has been investigated in many diseases, such as inflammatory diseases, skin diseases and cancer. DMF is thought to have immunomodulatory properties without causing apparent immunosuppression [79]. Although the precise mechanism of action is incompletely characterized, DMF can modulate the Nrf2/Keap1 signaling pathway by targeting Keap1 [79]. DMF, as an electrophile, can induce covalent changes of some cysteine residues of the Keap1 protein, especially cysteine 151, which alters the conformation of the Keap1 protein, leading to diminished Keap1–Nrf2 interaction [80]. DMF could also stimulate Nrf2 activation by enhancing the PI3K/AKT/GSK-3β pathway [81].

Today, DMF is approved as an oral treatment for psoriasis and relapsing–remitting multiple sclerosis (RRMS) before initiating biological therapy. Its safety and tolerability proved to be acceptable in clinical trials; nevertheless, DMF causes adverse gastrointestinal effects and hematological disorders, such as leucopenia [82]. In human renal mesangial cells, DMF upregulated Nrf2 expression and its transcriptional targeting genes HO-1 and NQO1 in a time-dependent manner. Case reports have described the positive effects of the use of DMF in cutaneous lupus erythematosus (CLE) [83].

### 3.2. Sulforaphane

Sulforaphane (SFN) is a sulfur-containing isothiocyanate that is widely present in cruciferous plants and is well-known as an activator of Nrf2 [84]. Upon entrance of SFN into mammalian cells, it is conjugated with GSH in a reaction catalyzed by glutathione S-transferase (GST). Thereafter, it enters the mercapturic acid pathway until it is converted into dithiocarbamates, and eventually is excreted through the urine [85,86]. Traditionally, SFN has been used as a diet supplement [87]. In humans, moderate doses of SFN appear to be rapidly metabolized [88]. However, it is worth noting that high doses of SFN exerted cytotoxic and pro-oxidative effects by boosting DNA damage, which could be related to the overreaction of Nrf2-mediated Kruppel-like factor 9 (Klf9) expression and suppression of peroxiredoxin 6 (Prdx6) (in Nrf2/Keap1 axis), inducing cell apoptosis [89].

SFN is characterized by the presence of electrophilic groups, for instance, isothiocyanate, which has high chemical reactivity. In the cytosol, the isothiocyanate group of SFN promotes the modification of the cysteine sensor on Keap1 and induces disruption of the Nrf2–Keap1 complex, increasing the transcription of Nrf2 into the nucleus [84]. Furthermore, SFN inhibits glycogen synthase kinase 3β (GSK3β) activity and boosts the nuclear translocation of Nrf2, which is stabilized by blocking the proteasome-reliant degradation [90,91]. Currently, there are no relevant studies on the effects of SFN in SLE patients in vitro or in vivo.

However, SFN is used in clinical trials for other diseases, including cancer, COPD, asthma, fatty liver disease and diabetes. The use of SFN in daily practice is still limited due to poor druggability [92].

### 3.3. Artemisinin and Its Derivatives

Artemisinin (ARS), a hemiterpene lactone endoperoxide, is the main active ingredient of the traditional Chinese herb Artemisia annua, which has various derivatives, including dihydroartemisinin (DHA), artesunate (ART), artemether and arteether [93]. ARS and its derivatives are widely recognized as the most effective drug in malaria treatment, and several studies have shown that it also benefits beyond its anti-malarial effects, such as in cancer and inflammatory and immune-mediated diseases [94,95]. Other studies stated that ARS and its derivatives protect against renal injury by regulating inflammation and OS and stabilizing the autophagy function of podocytes [93,96]. An ARS family compound, namely newly synthesized compound 2′-aminoarteether (β) maleate (SM934), has been used in clinical trials as a novel therapeutic agent next to the standard of care in SLE, resulting in synergistic immune suppressive effects [97]. Different studies have shown that ARS and its derivatives are safe, with no severe adverse effects; however, reports on clinical trials are not present. Commonly reported less severe adverse effects include nausea, vomiting, diarrhea, transient transaminases elevation and mild rash, which are usually self-resolving [93,98].

### 3.4. Triterpenoids and Bardoxolone Methyl

Triterpenoids are natural compounds that represent one of the most structurally diverse groups of chemicals, which are secondary metabolites of peels, leaves, stems and wax-like coatings of various fruits, such as olive leaves, olives, camellia sinensis and olea europaea [99,100]. Although the biological significance of these compounds is not completely clear, so far, it has been shown that they might have anti-cancer, anti-inflammatory, anti-oxidative, anti-viral, anti-bacterial and anti-fungal properties [101].

Bardoxolone methyl (CDDO-Me) and its analogs (e.g., CDDO-imidazolide (CDDO-Im)) are semi-synthetic triterpenoids derived from oleanolic acid, which can be obtained in high yields from olive pulp residue [102]. CDDO-Me and its analogs contain electrophiles and inhibit Nrf2–Keap1 protein–protein interaction through covalent modification of the critical cysteine residues in Keap1 [103]. In addition, the structure of CDDO-Me mimics the cyclopentenone prostaglandins (such as 15-deoxy-Δ12, 14 prostaglandin J2), which are the most powerful endogenous activators of the Nrf2–Keap1 complex [104].

In clinical studies, CDDO-Me has been mainly applied in treating malignancy, chronic kidney disease (CKD), type 2 diabetes mellitus (T2DM) and diabetic nephropathy, showing side effects including transient, reversible elevations in aminotransferases levels, hypomagnesaemia, gastrointestinal disorders and decreases in body weight, while no clinical study on LN has been performed [105,106,107]. In a double-blind phase 3 clinical trial in patients with CKD and T2DM, the participants were randomly administered either a daily placebo or 20 mg of CDDO-Me. The study was suddenly terminated around 9 months because severe adverse events were noticed in 33% of the patients who were treated with CDDO-Me and higher mortality of patients regardless of any etiology was reported in the experimental group [108]. This investigation of course raised concerns for the use of CDDO-Me in the treatment of kidney disease. But as mentioned, no clinical trials in SLE have been performed.

### 3.5. Polyphenols

Polyphenols are secondary metabolites of plants, present in fruits, vegetables and spices, known for their antioxidant, anti-inflammatory and immunomodulatory activities. They have attracted much attention due to their nutritional and possible medicinal value [109]. Phenolic compounds have at least one aromatic ring with one or more hydroxyl groups [110]. Regarding the number and arrangement of the carbon atoms, polyphenols can be classified into four major groups: Flavonoids (Flavan-3-ols: Epigallocatechin 3-gallate (EGCG); Theaflavin), Stilbenes (resveratrol, pterostilbene), Phenolic acids (salicylic, Syringic acid, caffeic, ferulic) and Lignans (Pinoresinol) [111]. It is known that phenolic compounds are the most affluent in human diets among the dietary antioxidants, and the long-term consumption of diets rich in polyphenols protecting against cancer, diabetes, nephropathy and cardiovascular disease has been reported [112]. Polyphenols are either acetylene compounds or can be metabolized to reactive electrophiles capable of alkylating cysteine thiols in Keap1 [113]. A randomized, double-blinded clinical trial of CKD patients on hemodialysis has demonstrated the beneficial effects of polyphenol supplementation on muscle and bone mass. On the contrary, another randomized double-blind clinical trial demonstrated that 500 mg of resveratrol supplementation for 1 month had no antioxidant or anti-inflammatory effect in non-dialyzed CKD patients. However, this trial used the supplement only for a short time and at a fixed dose [114,115]. Additionally, a randomized trial of a polyphenol-enriched diet illustrated that patients with a diet of standard protein restriction (0.8 g/kg) had a higher albumin–creatinine ratio, podocyte apoptosis, and more frequent renal replacement therapy and mortality than patients on polyphenol-enriched diets [116].

Studies on the biological activity of polyphenols have primarily been performed in mice models, including olive oil, EGCG (the major constituent of green tea), resveratrol (RSV), salvianolate acid (extraction of salvia miltiorrhiza, a Chinese traditional herb), curcumin (an active ingredient in the curry spice turmeric), α-mangostin (the major constituent of mangosteen), mangiferin (main source of mango) and punicalagin (the major constituent of pomegranate). However, a few studies were performed in human cells or patients, which are listed below.

Aparicio-Soto M et al. found that extra virgin olive oil (EVOO) and its extracts decreased cytokine production and T cell activation, and increased expression of IκB-α (preventing NF-κB activation), indicating beneficial effects of EVOO on PBMCs from SLE patients compared to HC [117].

EGCG has been reported to reduce the expression of different autoantigens at the mRNA and/or protein levels in an in vivo model of normal human primary epidermal keratinocytes, a cell type particularly affected in patients with SLE, leading to a decrease in the expression of several antinuclear autoantibodies [118].

In a recent review by Huo et al. [119], the effects of resveratrol on ameliorating immune disorders by inhibiting the overactivation of immune cells is described. Advances in research on the protective effects and potential mechanisms of RSV against SLE and its use as a promising therapeutic option for the treatment of SLE are shown. A study found that monocytes in the blood of patients with active SLE co-cultured with RSV significantly reduced the level of IL-10 in cell culture supernatant [120].

Another study demonstrated that both curcumin and vitamin D individually improved clinical outcomes and cytokine levels in SLE patients, while the combination of curcumin–piperine with vitamin D was the most effective in reducing disease activity, IL-6 levels and fatigue severity scale [121]. That curcumin could be used as a safe adjuvant therapy for LN patients was also investigated through a randomized, placebo-controlled study, which showed that short-term oral curcumin supplementation significantly reduced proteinuria, hematuria and systolic blood pressure in patients with relapsing or refractory LN without side effects [122].

There are no experimental data or clinical trial studies on the effects of mangostin, punicalagin, salvianolate acid and mangoferin in SLE or LN patients, but some effects of these compounds have been described in studies reviewing the effects of diets and nutrients [123,124,125,126].

## 4. Immunomodulatory Effects of Diet in SLE

Currently, awareness is growing that diet may affect immunomodulation, which could be of importance in the management of SLE [127]. A study investigating nutritional status and food intake in SLE patients demonstrated that around half of the patients were above normal weight, and one-tenth of the patients were classified as moderately to severely malnourished. There were not only fruits, vegetables and dairy products consumed below requirements; but also calcium intake was inadequate, and the consumption of oils and fats was high [128].

The consumption of specific nutrients may have a beneficial effect on patients with SLE not only due to direct effects on OS, inflammation and the immune system, but also to indirect effects on obesity and associated co-morbidities [129]. Moreover, diet and nutrient-adjuvant therapy rarely have side effects compared to standard therapy [130]. Therefore, there could be a possible adjunctive role for diet and nutrients in the management of SLE.

Monitoring nutritional status along with an adequate diet and implementing cognitive–behavioral therapy as well as counseling interventions revealed an improvement of SLE prognosis and mood and quality of life for patients [126]. A personalized diet that restricts calories and fat but is enriched with fiber, protein, polyunsaturated fatty acids (PUFAs)/mono-unsaturated fatty acids (MUFAs) (mainly ω-3 and ω-6), minerals (selenium, calcium, zinc, iron and copper) and a variety of vitamins for SLE patients should be proposed [131,132,133]. Furthermore, studies have suggested that the traditional Mediterranean diet, which is a plant-based diet with small amounts of meat, wine and olive oil, might also be beneficial in SLE by diminishing OS and inflammation [59,134].

## 5. Side Effects of Nrf2 Activation

Direct disruption of the Nrf2–Keap1 complex, promoting Nrf2 translocation from cytosol to the nucleus or preventing its degradation to activate Nrf2, serves as a novel therapeutic strategy for enhancing antioxidant defense, which has been proven to be effective in many diseases; however, aberrant Nrf2 activation may lead to unanticipated side-effects. On the one hand, it is hypothesized that in malignant cells, mutations in Keap1 can result in permanent Nrf2 activation, which is beneficial to tumors [135]. Cancer patients with mutations in Keap1 or Nrf2 have a worse prognosis than patients without such mutations [136]. On the other hand, the indiscriminate pursuit of Nrf2 activation may not consistently yield advantageous results. ROS promotes the cell-surface disulfide-containing protein formation, aiding in protein construction and signal transduction. Excessive Nrf2 reduction may inhibit ROS function, driving reductive stress, and overreduction destroys the integrity of protein function and inhibits cellular growth factor receptors, thereby reducing cellular metabolism [137]. Low-dose Nrf2 agonists, for instance, bardoxolone methyl dh404, may attenuate atherosclerosis and reduce proteinuria, mesangial expansion and glomerular damage in diabetic nephropathy, although high-dose Nrf2 agonists may result in the expression of pro-inflammatory mediators such as monocyte chemoattractant protein-1 (MCP-1) and NF-κB [138]. Therefore, it is of importance to cautiously balance Nrf2 activation to avoid potential adverse outcomes, and optimizing the concentration of Nrf2 agonists is necessary. Further studies are needed to establish safe and effective guidelines for Nrf2-targeting therapies.

## 6. Summary and Conclusions

In summary, Nrf2 is an essential transcription factor for OS response that plays a crucial role in many processes, including inflammation, immunological responses, metabolism and mitochondrial physiology. The effects of the Nrf2 pathway in SLE are complex and pleiotropic. However, there is some contradiction in the treatment of SLE regarding this pathway. A more elaborate understanding of the precise molecular targeting mechanisms involved is an absolute prerequisite for the development of personalized redox medicine approaches and there is still a significant gap in translating these findings into clinical application. The current research on Nrf2 in the context of SLE primarily comprises cellular and animal studies with limited clinical investigations. There have been only a few clinical trials related to Nrf2 agonists in SLE patients, which mainly focused on symptoms or altered laboratory markers rather than comprehensive clinical outcomes. Moreover, in order to avoid unpredictable serious adverse events, it is important to ensure that doses of Nrf2 agonists are within safe boundaries. More research on Nrf2 and its extensive therapeutic application as an add-on to the standard treatment of SLE should be performed, as it might be a promising approach to enhance antioxidant expression, decrease ROS production, and suppress inflammatory response. Furthermore, the question of which compound is the best to activate Nrf2 is not answered yet. In the meantime, a healthy diet enriched in fiber, antioxidants and PUFAs/MUFAs should be advised for SLE patients.

In conclusion, regulating Nrf2 pathways has potential benefits in patients with SLE. The complexities of Nrf2 signaling and its interactions with various pathways highlight the need for continued exploration. By diving deeper into these mechanisms, novel approaches might be identified to improve patient outcomes and quality of life.

## Figures and Tables

**Table 1:** Effect of Nrf2 activators in mice model of LN.

Nrf2 Inducer	Compounds Derivation Sources	Mouse Model	Effects	Kidney Outcome	Ref.
EGCG	Green tea	NZB/W F1 mice	↑NQO1, HO-1, and GPx; ↓NF-κB	↓urinary protein/Cr ratio and serum BUN, Cr; ↓severe renal lesions, ↓IgG deposits in kidneys	Tsai et al., 2011 [56]
Antroquinonol	Antrodia camphorata	NZB/W F2 mice, ASLN model	↑GPx; ↓ROS	↓urinary protein/Cr ratio, Hematuria and serum BUN, Cr; ↓severe renal lesions.	Tsai et al., 2012 [57]
Citral	Litsea cubeba	NZB/Wf1 mice, ASLN model	↓NF-κB, ROS	↓urinary protein/Cr ratio, serum BUN, Cr; ↓severe renal lesions; ↓renal apoptosis	Ka et al., 2015 [58]
Sulforaphane	All cruciferous vegetables	Pristane-treated mice	↑NQO1	↓urinary albumin/Cr ratio, index of glomerular pathology, beneficial effects disappeared in Nrf2 knockout mice	Jiang et al., 2014 [48]
EVOO	Olives	BALB/c mice, pristane-treated	↑HO-1, IκBα; ↓pSTAT-3	↓proteinuria, renal damages, foci of inflammation in interstitium	Aparicio-Soto et al., 2016 [59]
Sulforaphane/DMF	All cruciferous vegetables/ fumaric acid	BALB/c mice, pristane-treated	↑HO-1, NQO1; ↓NF-κB	↓proteinuria, fibronectin expression, renal inflammation, ameliorated renal histopathology with DMF	Ebihara et al., 2016 [60]
Melatonin	Pineal gland	BALB/c mice, pristane-treated	↑SIRT1; ↓NF-Κb	↓glomerular lesions, MMA was restored, comparable glomerular volume and area to controls	Bonomini et al., 2019 [61]
OL	Olives	BALB/c mice, pristane-treated	↑HO-1, IκB-α; ↓pSTAT-3, NF-κB, MAPK phosphorylation	↓interstitial fibrosis, renal damages	Castejon et al., 2019 [62]
DHA	Artemisia annua	BALB/c mice, pristane-treated	↑HO-1, NQO1	↓urine protein, serum BUN, Cr, renal damages, ROS levels in kidneys, foot process effacement of podocyte	Li et al., 2019 [63]
Baicalein	Scutellaria baicalensis Georgi	BALB/c mice, pristane-treated	↑GPx, HO-1, NQO1; ↓NF-κB	↓urine protein, foot process effacement of podocyte, ROS levels in kidneys; glomerulonephritis remission	Li et al., 2019 [64]
Artemisinin derivatives	Artemisia annua	NZW × BXSB mice	↑HO-1, SOD, GPx, CAT; ↓MPO; ↑nuclear Nrf2 in vitro, ↓Cytosol Nrf2 in vitro; beneficial effects disappeared in Nrf2 knockout cells	↓proteinuria, urinary albumin/Cr, serum BUN, tubulointerstitial fibrosis, glomerular lesions	Lin et al., 2021 [65]
Jieduquyuziyin prescription	Mixed herb extracts	MRL/lpr mice	↑SOD	↓index of renal damage, urinary protein, urinary albumin/Cr ratio, ROS, MDA levels in kidneys	Du et al., 2022 [66]
Esculetin	Hydrangea paniculata	MRL/lpr mice	↑HO-1, NQO1; ↓Keap1, NF-κB	↓urinary albumin/Cr ratio, BUN/Scr ratio, serum BUN, Cr, glomerular damage score, protein cast, tubular dilation and immune cells infiltration	Zhang et al., 2022 [67]
OLE	Olive oil	BALB/c mice, pristane-treated	↑HO-1, IκB-α; ↓MAPK phosphorylation, pSTAT-3, NF-κB	↓glomerulosclerosis, extraglomerular alterations, immunocomplexes deposits	Montoya et al., 2023 [68]
OLA	Olive oil	BALB/c mice, pristane-treated	↑ IκB-α, ↓JAK/STAT, MAPK phosphorylation, NF-κB	↓renal histopathological alterations, immunocomplexes deposits	Muñoz-García et al., 2023 [69]

↑, upregulation or increase; ↓, downregulation or decrease. Abbreviations: MMA: intraglomerular mesangial matrix; EVOO: Extra virgin olive oil; EGCG: Epigallocatechin-3-gallate; DMF: Dimethyl Fumarate; OL: Oleocanthal and oleuropein; DHA: Dihydroartemisinin; OLE: Oleocanthal; OLA: Oleacein; MDA: Malondialdehyde; BUN: Blood urea nitrogen; Scr: Serum creatinine. For other abbreviations, see figures.

## Data Availability

No initial data were used for this article.
